# When diabetic ketoacidosis becomes a neurological emergency: cerebral edema in a child with new-onset type 1 diabetes mellitus - a case report and narrative review

**DOI:** 10.3389/fendo.2026.1921269

**Published:** 2026-09-28

**Authors:** Mateusz Okonek, Anna Paczkowska, Kinga Drzewińska, Patrycja Rozwadowska-Kunecka, Anna Wołoszyn-Durkiewicz, Radosław Owczuk, Małgorzata Myśliwiec

**Affiliations:** 1 Student Scientific Circle of Pediatrics, Diabetology and Endocrinology, Faculty of Medicine, Medical University of Gdańsk, Gdańsk, Poland; 2 Department of Pediatrics, Diabetology and Endocrinology, University Clinical Center in Gdańsk, Gdańsk, Poland; 3 Department of Pediatrics, Diabetology and Endocrinology, Faculty of Medicine, Medical University of Gdańsk, Gdańsk, Poland; 4 Department of Anesthesiology and Intensive Therapy, Faculty of Medicine, Medical University of Gdańsk, Gdańsk, Poland; 5 Department of Anesthesiology and Intensive Therapy, University Clinical Center in Gdańsk, Gdańsk, Poland

**Keywords:** acute complications of diabetes, case report, cerebral edema, diabetic ketoacidosis, hyperglycemia, type 1 diabetes mellitus

## Abstract

**Background:**

Diabetic ketoacidosis (DKA) is a life-threatening complication of type 1 diabetes mellitus (T1D), potentially leading to severe outcomes, including cerebral edema (CE). The case describes an eight-year-old child with previously undiagnosed T1D complicated by DKA, and subsequent development of CE. It is followed by a short literature review including other pediatric cases of clinically overt CE.

**Case presentation:**

A boy was admitted to the outpatient clinic with a several-day history of vomiting, abdominal pain and headache, leading to a presumptive diagnosis of gastrointestinal infection. The following day, the patient’s condition deteriorated. Despite a positive family history of T1D (the patient’s father), his blood glucose level was measured for the first time by the emergency medical service (25.6 mmol/L (460 mg/dL)). Further evaluation revealed DKA secondary to previously undiagnosed T1D. On admission, the boy was in severe general condition. Severe DKA was confirmed (pH 6.88), and appropriate treatment was implemented, resulting in temporary clinical stabilization. However, on the second day of hospitalization, the patient’s condition deteriorated again. He was somnolent, with fluctuating consciousness and nuchal rigidity. An increase in intracranial pressure (ICP) was suspected. Optic nerve ultrasonography revealed increased optic nerve sheath diameters, also suggestive of raised ICP. Anti-edema therapy with mannitol was initiated based on clinical suspicion, resulting in clinical improvement. Dexamethasone and furosemide were administered as adjunctive therapy. Subsequent CT findings supported the diagnosis of CE. The patient was discharged after 10 days in good general condition.

**Conclusions:**

DKA can be a diagnostic challenge for clinicians due to its nonspecific symptoms. Early signs suggesting this condition should not be underestimated, and bedside blood glucose should be measured early in children presenting with unexplained vomiting, weight loss, tachypnea, dehydration or altered consciousness. Close monitoring for complications is critical during treatment, especially for CE, one of the most serious and potentially life-threatening consequences of DKA.

## 1 Background

Diabetic ketoacidosis (DKA) is a life-threatening manifestation of type 1 diabetes (T1D) potentially leading to severe adverse outcomes, including cerebral edema (CE) (1). CE is considered the most severe complication of DKA and carries a particularly high mortality risk in children affected with DKA (2). Studies have shown that CE occurs in about 7 per 1000 episodes of DKA and is more frequently observed in patients at the time of initial diagnosis (3).

Despite its relatively low incidence, CE accounts for the majority of diabetes-related deaths in children with DKA (1). Mortality rates among patients who develop CE are estimated to range from approximately 20% to 25%, while a significant proportion of survivors experience long-term neurological sequelae such as cognitive impairment, memory deficits, or motor dysfunction (4).

The pathophysiology of DKA-related CE reflects a complex interaction among cytotoxic, vasogenic, and osmotic mechanisms, resulting in elevated intracranial pressure (ICP) and subsequent neuronal injury. Several pathophysiological pathways have been proposed, including cerebral hypoperfusion followed by reperfusion injury, disruption of the blood–brain barrier, osmotic fluid shifts associated with rapid changes in serum osmolality, and inflammatory processes that increase cerebral vascular permeability (5). Both disease-related factors and treatment-related variables have been implicated as potential contributors to the development of CE. Despite an extensive number of studies, precise underlying mechanisms remain incompletely understood, highlighting the need for further research.

Multiple past investigations have focused on identifying risk factors for CE and our understanding in this area continues to evolve. Recent studies estimate that a higher initial serum urea nitrogen concentration and lower initial partial pressure of carbon dioxide are linked to the development of CE (1, 6). Although some guidelines suggest that early childhood may be associated with cerebral edema in DKA, published evidence does not consistently support young age as an independent risk factor (7).

Currently, CE in pediatric patients with DKA most often presents in a subclinical form, with only subtle neurological symptoms and abnormalities that are frequently not detected on computed tomography (CT) (8). Improvements in the early diagnosis and management of T1D may have been associated with a reduction in the frequency and severity of DKA at presentation, which is a major risk factor for CE (6). Because of the rapid progression and potentially fatal outcome of CE, early detection of warning signs - such as headache, altered mental status, vomiting, or sudden deterioration in neurological status – is crucial for timely intervention. Prompt treatment, including the administration of osmotic agents such as mannitol or hypertonic saline, is essential to reduce the mortality rate and prevent permanent neurological injury (9).

We present a case of CE in the course of DKA with very low initial pH (6.88) in an eight-year-old child with new-onset T1D, whose initial symptoms were misinterpreted, resulting in a delayed diagnosis and subsequent development of this complication. The report highlights the importance of early recognition and management of this rare but potentially fatal complication.

## 2 Case presentation

An 8-year-old boy was transferred by air ambulance from a regional hospital to a tertiary University Clinical Center due to newly diagnosed T1D presenting with severe DKA. The patient had a one-week history of abdominal pain, headache, and vomiting that began two days before initial medical evaluation. A few days before symptom onset, the boy had been in close contact with a friend who was subsequently diagnosed with rotavirus infection. Family history was positive for T1D in the patient’s father and autoimmune hypothyroidism in the patient’s mother. At that time, he was evaluated at the primary care service, where a gastrointestinal infection was suspected. During the night, he developed tachypnea, dyspnea, and profound weakness. Emergency medical services were called, and the capillary blood glucose was measured for the first time, revealing a level of 25.6 mmol/L (460 mg/dL). The patient was subsequently transferred to the pediatric intensive care unit (PICU).

The parents denied polyuria, polydipsia, or nocturia. However, a 4-kg weight loss over the preceding month was reported. On presentation, the boy was in severe general condition, with a Glasgow Coma Scale (GCS) score of 13. He was somnolent, but responsive to verbal stimuli. Physical examination revealed Kussmaul respiration, tachycardia (134/min), blood pressure 121/55 mmHg, respiratory rate 30/min, oxygen saturation 100%, temperature 36.6 °C, and prolonged capillary refill time (>2 s). The patient’s characteristic Kussmaul breathing is presented in **Supplementary Material** (**Audio S1**). Anthropometric measurements showed a height of 1.39 m (75th-90th percentile), weight of 32 kg (25th-50th percentile, adjusted for height age), and body mass index 16.56 kg/m2 (50th-75th percentile). The estimated fluid deficit was approximately 10%.

Laboratory findings detailed in **Table 1** confirmed severe DKA with profound metabolic acidosis (pH 6.88, HCO_3_^-^ 4 mmol/L, pCO_2_ 2.8 kPa (21.1 mm Hg), base excess -29.2 mmol/L) and marked hyperglycemia, with elevated glycated hemoglobin (HbA1c) at 13.1%. Corrected sodium levels (calculated using the Katz formula: corrected sodium (mmol/L) = measured sodium (mmol/L) + 0.016 × [measured glucose (mg/dL) − 100]) (9) ranged from 132.1 to 137.3 mmol/L. During treatment, serum sodium remained relatively stable. Initial management included fluid resuscitation with 0.9% sodium chloride administered at a rate of 10 mL/kg/h, followed by continuous intravenous insulin infusion at 0.02 units/kg/h (the infusion rate was gradually increased). Further fluid therapy (0.9% sodium chloride and a multi-electrolyte solution) and insulin infusion were administered according to the institutional DKA protocol, with doses adjusted according to serial clinical and laboratory assessments. During treatment, the boy required potassium and phosphate supplementation. Thromboprophylaxis with enoxaparin at a dose of 20 mg (once daily, for six consecutive days) was also administered. Considering the patient’s clinical condition and laboratory findings, an 8.4% sodium bicarbonate solution (a single 40-mmol dose, infusion duration: 1 hour) was included in the initial management.

**Table 1:** Serial laboratory values during hospitalization.

Parameter [normal range]	Day 1 (ER/PICU)^#^	Day 2 (PDW/PICU)^$^	Day 3 (PDW/PICU)^$^
Glucose [3.9-5.5 mmol/L (70–99 mg/dL)]	26.8 (482)	6.6 (119.4)	–
HbA1c [<6.5%]	13.1	–	–
Bicarbonate [21–32 mmol/L]	4	12.5	25
pCO_2_ [4.7–6 kPa (35–46 mm Hg)]	2.8 (21.1)	3.5 (26.2)	5.4 (40.5)
pH [7.35-7.45]	6.88	7.3	7.4
Base excess [(-2)-3 mmol/L]	-29.2	-12.6	0.9
Sodium [136–145 mmol/L]	126	137	133
Potassium [3.5-5.1 mmol/L]	4.7	3.2	4.2
Blood urea nitrogen [2.5–6 mmol/L (7-16.8 mg/dL)]	5 (14)	2.5 (7)	–
Serum osmolality [280–295 mOsm/kg H2O]	294.6	316*	306*
Ketones in urinalysis [-]	+++	+++	++
Glucose in urinalysis [-]	++++	++++	+

HbA1c, glycated haemoglobin; pCO_2_, partial pressure of carbon dioxide; ER, Emergency Room; PICU, Pediatric Intensive Care Unit; PDW, Pediatric Diabetology Ward.

*after mannitol administration; directly measured.

^$^ arterial blood gas.

^#^ capillary blood gas.

After approximately 24 hours, metabolic parameters improved (**Table 1**), and the patient was transferred to the pediatric diabetology ward equipped for continuous cardiorespiratory monitoring and regular clinical and laboratory reassessment. Immediately prior to transfer, the patient showed clear clinical improvement, with stable respiratory and cardiovascular parameters. Resolution of DKA was assessed using standard biochemical and clinical criteria, including acid–base status, anion gap, serum electrolytes, and clinical improvement. At the time of transfer from the PICU, the patient’s serum chloride concentration was 115 mmol/L, serum sodium was 137 mmol/L, bicarbonate was 12.5 mmol/L, and the anion gap was 9.5 mmol/L. Arterial blood gas analysis demonstrated normalization of pH (7.30), while the base excess remained −12.6 mmol/L.

Along with metabolic stabilization, the patient’s neurological status was improving, however, its assessment was partially limited by residual sedation. He exhibited fluctuating verbal responsiveness, failed to regain full alertness, and intermittently followed simple commands. On admission to the pediatric diabetology ward, he also reported a persistent frontal headache. Due to ongoing concerns, optic nerve sheath diameter (ONSD) was assessed by point-of-care ultrasonography using a Canon Xario 200G ultrasound system equipped with a high-frequency linear-array probe. The examination was performed with the patient in the supine position, the probe was placed gently over the closed eyelid, with a generous amount of ultrasound gel applied to avoid direct pressure on the globe. ONSD was measured 3 mm posterior to the globe, in accordance with standard technique, with several measurements obtained for each eye and averaged. The examination was performed by a second-degree specialist in pediatrics with extensive experience in ultrasonography, who was not blinded to the clinical suspicion of raised ICP, as the priority in this acute clinical setting was to ensure the most accurate and timely assessment in the patient’s best interest. Using an age-appropriate diagnostic threshold of 5.75 mm, previously validated in pediatric patients (10), the ultrasound revealed increased ONSD (6.5 mm in the right eye and 7.9 mm in the left eye) with bilateral elevation of the optic discs. These findings, in conjunction with the clinical presentation, were consistent with CE, prompting administration of the first dose of mannitol and urgent ophthalmological consultation. Fundoscopic examination, performed urgently at the bedside, did not reveal papilledema. A neurological consultation was also obtained, and examination identified somnolence, hypotonia, neck stiffness, and a positive bilateral Babinski sign, raising suspicion of increased ICP. Eventually, non-contrast head CT showed features consistent with CE (**Figure 1**). Clinically apparent cerebral edema was recognized approximately 24 hours after treatment initiation.

**Figure 1:**
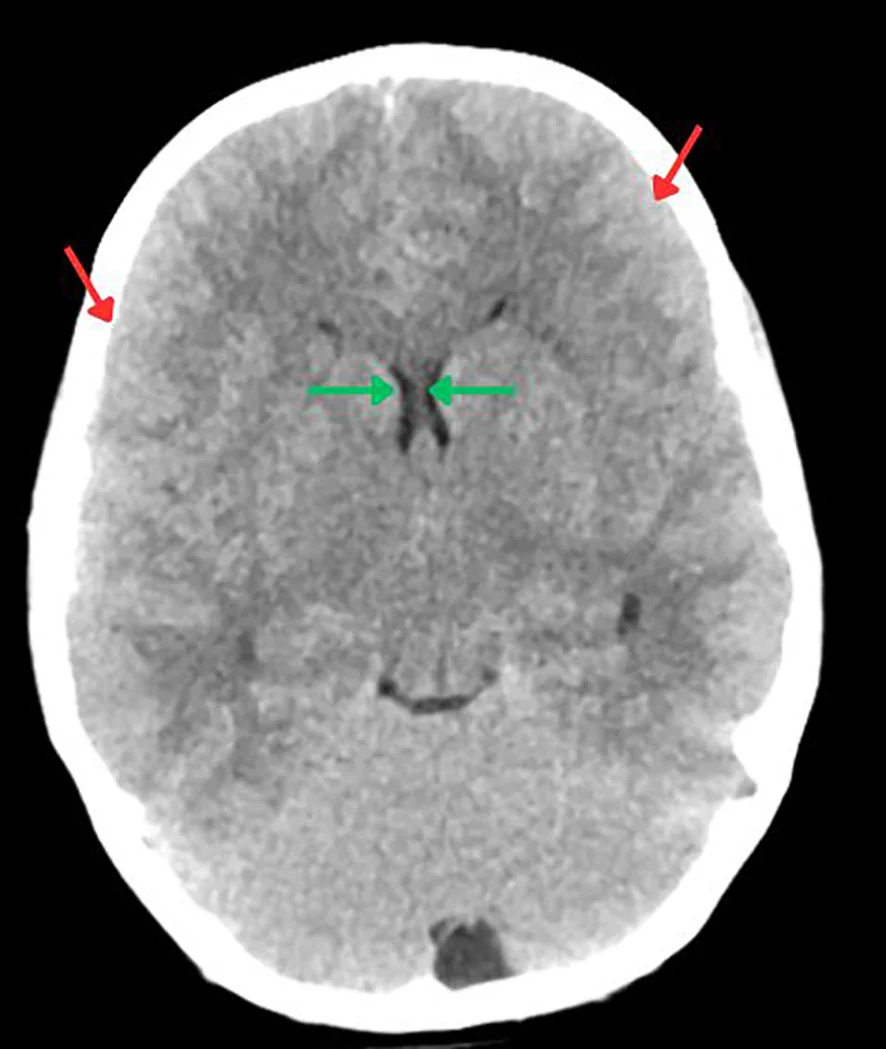
Brain computed tomography (CT) scan at the level of lateral ventricles, showing bilateral sulcal effacement (red arrows) and narrowing of the ventricular system (green arrows), representing radiological signs consistent with cerebral edema. The CT scan was performed approximately 24 hours after treatment initiation.

Hyperosmolar therapy was initiated with mannitol (two doses, each approximately 0.5 g/kg, with the second dose given 3 hours after the first; a total of 250 mL of 15% solution). Dexamethasone (2 mg intravenously twice daily; total dose, 4 mg) and furosemide (5 mg intravenously four times daily; total dose, 20 mg) were administered as adjunctive, case-specific therapies at the discretion of the treating team, based on clinical judgment and local practice.

Due to the diagnosis of CE, the patient was transferred back to the PICU, with continuation of treatment. By the third day of hospitalization (approximately 48 hours after admission), the acid–base status had fully normalized, with a pH of 7.4, a base deficit of 0.9 mmol/L, and a bicarbonate concentration of 25 mmol/L. Following normalization of the acid–base parameters, intravenous insulin therapy was discontinued and the patient was transitioned to subcutaneous insulin therapy. Intravenous fluid therapy was also discontinued approximately 48 hours after admission. Gradual clinical improvement was observed, with resolution of neurological symptoms. The patient was subsequently transferred back to the pediatric diabetology ward and discharged in good general condition after 10 days of hospitalization. The patient’s clinical course, diagnostic evaluation, and management are illustrated in **Figure 2**.

**Figure 2:**
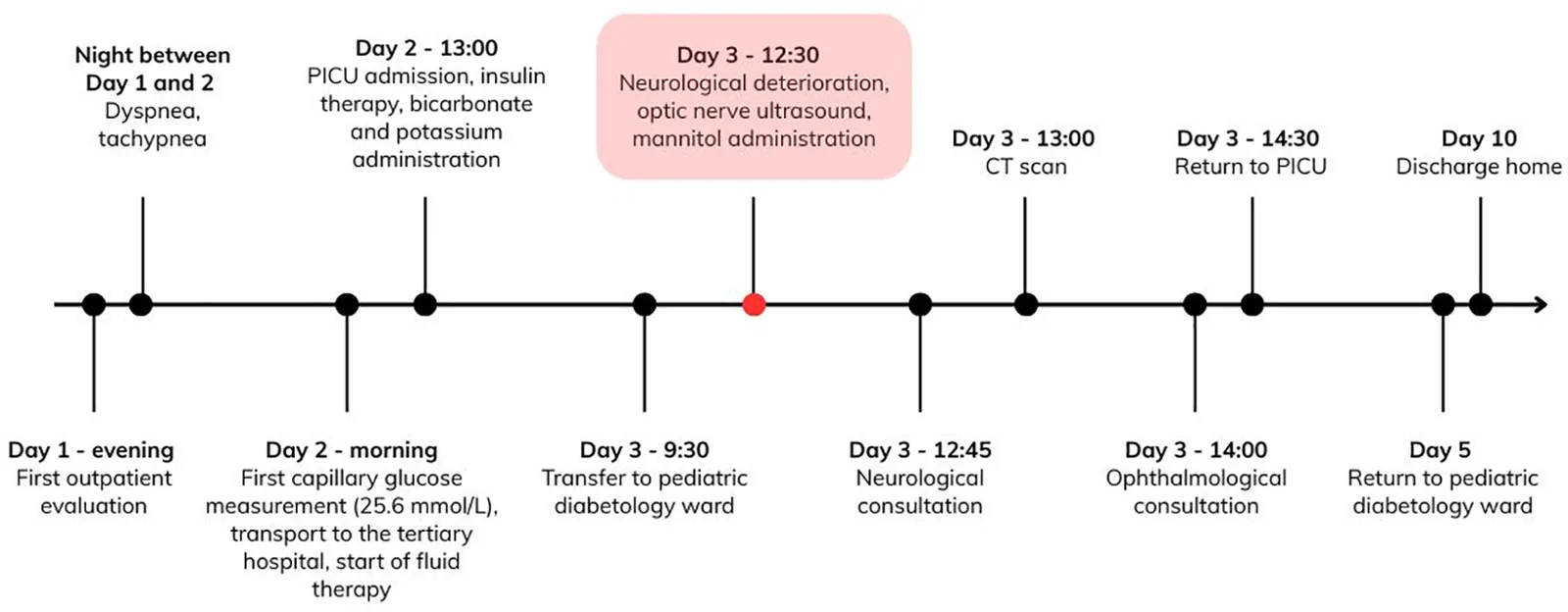
Timeline illustrating the patient’s clinical course, diagnostic evaluation, and management. PICU, Pediatric Intensive Care Unit; CT, computed tomography.

Neurological examination performed at a follow-up visit 2 months later showed good general condition, normal verbal contact, and no reported complaints. The patient was fully oriented to self and surroundings. Cranial nerve function was symmetric and normal, muscle strength was normal and symmetric, there were no signs of ataxia, sensation was normal, meningeal signs were negative, and gait was normal. No focal neurological or meningeal signs were present.

The patient has continued regular follow-up at the diabetes outpatient clinic. At the most recent follow-up visit in May 2026 (approximately 1.5 years after discharge), physical examination was unremarkable. The patient uses personal insulin therapy with a Medtronic 780G insulin pump and a Guardian 4 continuous glucose monitoring sensor, operating in hybrid closed-loop mode. Glycemic control was very good: mean glucose concentration 135 mg/dL, glucose management indicator 6.5%, coefficient of variation 30.6%, time in range 83%, time above range 16%, and time below range 1%. Total daily insulin dose was 22.1 units, including a basal dose of 10.9 units. HbA1c was 6.7%.

Based on the available clinical follow-up data, the patient has shown no evidence of permanent neurological sequelae to date, and his overall health status and metabolic control of diabetes remain excellent.

## 3 Discussion

In recent years, the incidence of T1D among children and adolescents has substantially increased. Clinical manifestations vary widely, sometimes resulting in diagnostic uncertainty and clinical challenges. This, unfortunately, may lead to delayed diagnosis, which can contribute to the development of several complications, such as DKA. A systematic review performed by Rugg-Gunn et al. showed that prompt diagnosis at T1D onset was associated with reduced risk of DKA and its sequelae (11). Unfortunately, DKA remains the initial manifestation of T1D, with incidence varying considerably across geographic regions (12, 13). To reduce its incidence, some countries have already implemented early screening programs based on the presence of islet autoantibodies (14–16). Such screening enables to detect early-stage T1D, and, consequently, to closely monitor the patients at the highest risk of the progression to clinical T1D. A significant reduction in the incidence of DKA among these populations was observed, along with the decrease in the incidence and the duration of hospitalizations (17, 18). Furthermore, several studies emphasized the value of tailored education and public awareness campaigns in reducing diagnostic delay (19, 20). The presented case highlights the importance of a thorough diagnostic evaluation to avoid delayed or incorrect diagnosis of potentially life-threatening conditions.

Nevertheless, the presented case highlights that thorough history-taking and careful clinical assessment remain essential in everyday practice. In particular, the positive first-degree family history of diabetes, combined with progressive weight loss, vomiting, headache, and deteriorating general condition, may serve as important diagnostic clues prompting earlier blood glucose testing, even when the initial presentation is nonspecific and alternative explanations, such as rotavirus infection, are considered. Another important aspect was the neurological course, which did not improve despite gradual increase in metabolic parameters. Thus, persistent impairment of consciousness should always prompt careful reassessment, as altered mental status may reflect evolving cerebral injury rather than metabolic disturbance alone. Finally, long-term follow-up remains an important element of care for children recovering from DKA-related CE, as subtle neurological sequelae may not become apparent immediately after hospital discharge. In our patient, subsequent follow-up visits, both neurological and diabetological, revealed no clinically evident deficits.

What is more, in our patient, optic nerve sheath ultrasonography was used as an adjunctive bedside tool supporting the diagnosis of CE. It should be emphasized that this technique is an indirect, bedside surrogate marker of elevated ICP rather than direct evidence of CE; in the present case, structural confirmation of CE was provided by head CT, while the abnormal ONSD findings served as an early, non-invasive signal prompting this further imaging. The discrepancy between the sonographic findings and the initial bedside fundoscopic examination in our patient warrants comment. Optic nerve sheath enlargement and papilledema differ markedly in their temporal dynamics, sonographic changes may occur within seconds to minutes of a rise in ICP, whereas papilledema – resulting from axoplasmic flow stasis – typically develops over hours to days (21). In our patient, ONSD ultrasonography was performed first, given the markedly elevated values, urgent ophthalmologic consultation was requested and mannitol was administered concurrently. By the time the fundoscopic examination was performed, ICP may have already begun to decrease in response to treatment, and, given the urgent, bedside nature of the assessment, the examination was performed using a Fison indirect ophthalmoscope – a monocular instrument with limited magnification and no stereoscopic capability – which may have limited its sensitivity in capturing the full extent of optic disc changes at that time. This discrepancy illustrates the complementary, rather than interchangeable, nature of the two methods. Although ONSD measurements showed an interocular difference (right eye 6.5 mm, left eye 7.9 mm), both values substantially exceeded the applied diagnostic threshold, and neuroimaging revealed no focal orbital pathology accounting for a unilateral process; such asymmetry is attributed to the operator-dependent nature of the technique rather than true anatomical asymmetry (10, 22). While this technique may facilitate rapid bedside assessment, its diagnostic performance in pediatric DKA has not been fully established. Consequently, ONSD measurements should only complement the neurological examination and neuroimaging when clinically indicated.

The management of patients diagnosed with DKA primarily includes fluid and electrolyte replacement. Insulin therapy, administered at a dose adjusted to body weight, should be initiated approximately 1 hour after the start of fluid resuscitation. According to the current International Society for Pediatric and Adolescent Diabetes (ISPAD) guidelines, various fluid protocols can be used without increasing the risk of cerebral injury. In most cases, fluid administration combined with intravenous insulin infusion is sufficient to reverse metabolic acidosis (9). Consequently, bicarbonate administration remains controversial due to the risk of paradoxical cerebrospinal fluid (CSF) acidosis and potentially increased likelihood of cerebral injury (23). Paradoxical CSF acidosis is thought to result from increased carbon dioxide (CO_2_) production following bicarbonate administration, with subsequent rapid diffusion of CO_2_ across the blood-brain barrier (24). Earlier studies suggested an association between sodium bicarbonate administration during DKA management and CE in children (25, 26). However, a recent retrospective study by Patel et al. did not find the association between bicarbonate therapy and an increased risk of CE (27). Nevertheless, the Polish Diabetes Association guidelines allow the cautious use of bicarbonate only in exceptionally severe DKA (arterial pH < 6.9), when accompanied by other clinical circumstances, such as life-threatening hyperkalemia, compromised cardiac function or impending hemodynamic instability, which is consistent with international recommendations (9, 28). In our case, the patient’s severe clinical condition at presentation, characterized by tachycardia, tachypnea, and signs of impaired peripheral perfusion, together with a pH of 6.88, prompted the administration of sodium bicarbonate. This decision was made based on the clinical judgement of the treating intensive care team, who considered the patient to be at risk of hemodynamic instability.

Another important component of DKA management is thromboprophylaxis. According to the 2022 ISPAD Clinical Practice Consensus Guidelines, pharmacological thromboprophylaxis may be considered in patients at high risk of thrombotic complications (9). A retrospective assessment identified several established risk factors for venous thromboembolism in this patient, including critical illness requiring admission to the PICU, the presence of a central venous catheter in the right internal jugular vein, and prolonged immobilization secondary to severe cerebral edema. In addition, the guidelines emphasize that patients with diabetic ketoacidosis (DKA) are at increased risk of thromboembolic complications, including cerebral venous thrombosis. Following an individualized assessment of the potential benefits and risks, the treating team decided to initiate thromboprophylaxis with enoxaparin. Enoxaparin was administered subcutaneously at a dose of 20 mg once daily for six consecutive days. Anticoagulant therapy was monitored using routine coagulation parameters. No thromboembolic complications were observed during hospitalization.

Among the numerous complications of DKA, CE deserves particular attention due to its severity and potentially fatal outcome. The literature review conducted by the authors confirmed that clinically overt CE has become relatively rare in recent years. The search was conducted in the PubMed database using the following search term: “diabetic ketoacidosis AND cerebral edema”. The literature search covered the 10 years preceding the review (from January 1st, 2015, to December 31st, 2025). The article type filter was set to “Case Reports”. Each article’s title and abstract was screened by a single reviewer. Articles describing clinically apparent CE in patients with DKA aged under 18 years were classified as eligible. Clinically apparent CE was defined as acute neurological deterioration occurring during DKA management, with or without neuroimaging confirmation, in cases where CE was diagnosed by the authors of the original article. During the second stage of the analysis, the full texts of these articles were reviewed. Based on this assessment, the decision regarding the inclusion or exclusion of each article was made by the reviewer. Articles not relevant to the topic were excluded, as well as articles describing patients aged ≥18 years and articles not written in English or without a full text available from official sources. No additional articles identified through hand-searching of references were included. If more than one case was described in the same report, these cases were analyzed separately.

Sixteen eligible published cases of clinically apparent CE were identified through the prespecified search strategy. They are presented in detail in **Table 2**, together with the case of our patient (8, 29–41). **Table 2** demonstrates the heterogeneity of initial findings, clinical manifestations, and management strategies. The age of the patients ranged from 6 weeks to 17 years. Nevertheless, among the published cases summarized, some recurring features can be found. CE was associated with new onset of T1D in 12/16 cases (75%), as well as in ours. Initial blood pH was reported in 14/16 cases. 9 of them were classified as severe DKA (defined as pH < 7.0), as the case reported by us. Most commonly, CE developed within the first few hours after treatment initiation, although in 1 case it was already present before therapy (37). Interestingly, neuroimaging findings were not reported in as many as 6/16 patients (38%), and not performed in one case (38), suggesting that the diagnosis was made based solely on clinical and laboratory findings. In all the remaining cases CT was performed, mostly revealing features indicative of CE, such as sulcal effacement and ventricular narrowing. Additional magnetic resonance imaging evaluation was reported in only one article (40). The use of bicarbonate during treatment, as in our patient, was also reported in only one other case (38). Anti-edema therapy was described in 12/16 analyzed articles, in 10 of which mannitol was administered (83%). The other frequently used agent was hypertonic saline (42%), in 3/16 reports as a first-line treatment (8, 40). Other treatment options included dexamethasone and furosemide (38), as well as in our patient, or prednisolone (37). Although corticosteroids are not recommended as standard therapy for DKA-related cerebral injury, dexamethasone has been administered in isolated case reports and in some institutional practices as an adjunctive treatment in severe cases managed in the PICU. However, evidence supporting its efficacy is lacking, and current guidelines do not recommend its routine use. In our patient, dexamethasone and furosemide were administered as supportive measures in addition to hyperosmolar therapy, based on the patient’s clinical condition and the treating team’s judgment. We acknowledge that neither corticosteroids nor loop diuretics are recommended as standard therapy for DKA-related cerebral injury, as evidence supporting their efficacy remains insufficient. Current recommendations identify mannitol or hypertonic saline as the cornerstone of hyperosmolar treatment in this setting (28). It is worth mentioning that the outcome in one report was fatal (31).

**Table 2:** Clinical characteristics of selected pediatric patients with diabetic ketoacidosis complicated by cerebral edema.

Study (author, year)	Sex	Age	New onset T1D	Time to diagnosis of cerebral edema (from treatment initiation)	Neuroimaging findings (CT/MRI)	Clinical presentation, GCS	Neurological abnormalities before DKA treatment	Intubation/ mechanical ventilation	Bicarbonate therapy	Initial blood pH	Treatment of cerebral edema	Outcome & follow-up duration
our case	M	8 years	Yes	24 h	CT: sulcal effacement, ventricular narrowing	Somnolence, fluctuating responsiveness, neck stiffness, GCS 13	No	No	Yes	6.88	mannitol 0.5 g/kg, dexamethasone, furosemide	Complete recovery; follow-up: at discharge (hospital day 10)
Kachuei et al., 2025 (40)	M	11 years	No	0.75 h	CT: mild sulcal effacement; MRI: diffuse edema, restricted diffusion, findings favouring infarction over encephalitis	Neurological deterioration, restlessness, bradycardia, hypertension, breathing disorders, GCS 3	Yes	Yes	No	7.19	300 mL hypertonic saline, 100 mL 20% mannitol	Complete recovery; follow-up: 1 month (no neurological sequelae)
Bassell et al., 2021 (39)	F	NA *(adolescent)*	Yes	NA *(early)*	CT: suspected edema, dural sinus thrombosis	Altered consciousness, minimal responsiveness, vomiting, GCS NA	Yes	Yes	NA	NA	mannitol, hypertonic saline	Complete recovery; follow-up: 10 months
Jeziorny et al., 2020 (38)	M	11 years	Yes	8 h	not performed	Headache, dilated pupils, loss of consciousness, respiartory arrest, GCS NA	Yes	Yes	Yes	6.93	mannitol, dexamethasone, furosemide, decompressive craniectomy	Complete recovery; follow-up: at discharge (hospital day 12)
Yoshizawa et al., 2019 (37)	F	10 years	Yes	before treatment initiation	NA	Altered consciousness, GCS NA	Yes	Yes	No	6.92	mannitol, prednisolone	Complete recovery; follow-up: NA
Carson et al., 2019 (41)	M	17 years	Yes	NA	CT: generalized edema	No improvement in consciousness, GCS NA	Yes	No	No	NA	mannitol (2 doses)	Complete recovery; follow up: at discharge hospital day 8)
Halloum et al., 2019 (36)	F	5 years	Yes	NA	CT: sulcal effacement, ventricular narrowing	Altered consciousness, GCS 10	Yes	Yes	No	6.92	mannitol 1g/kg, hypertonic saline 5 mL/kg	Complete recovery; follow-up: at discharge (hospital day 7)
Agarwal et al., 2018 (1) (8)	F	8 years	Yes	2 h	NA	Lethargy, headache, GCS 14	Yes	No	No	6.89	hypertonic saline 5 ml/kg	Complete recovery; follow-up: at discharge (hospital day 4)
Agarwal et al., 2018 (2) (8)	F	16 years	No	>2 h	NA	Headache, agitation, disorientation, GCS 14-15	No	No	No	6.97	hypertonic saline 5 ml/kg	Complete recovery; follow-up: at discharge (hospital day 3)
Baszyńska-Wilk et al., 2018 (34)	F	9 years	Yes	3–9 h	CT: diffuse edema, 13-mm hypodense lesion in left temporal lobe	Agitation, restlessness, upper limb spasms, altered consciousness, GCS 7	Yes	Yes	No	7.1	mannitol	Persistent motor neuropathy of the right lower limb; follow-up: NA
Ostojic et al., 2017 (35)	F	15 years	Yes	NA *(early)*	CT: diffuse edema	Headache, vomiting, lethargy → altered consciousness, coma, alpha coma on EEG, GCS 6	Yes	Yes	No	6.8	mannitol 0.5 g/kg (every 6 hours)	Recovery; follow-up NA
Day et al., 2017 (1) (33)	M	6 weeks	Yes	NA	NA	Altered consciousness, opisthotonus, seizures, GCS NA	Yes	NA	NA	7.16	NA	Persistent severe neurological disability; follow-up: 18 years
Day et al., 2017 (2) (33)	F	16 weeks	Yes	NA	NA	Altered consciousness, seizures, GCS NA	Yes	NA	NA	6.95	NA	Persistent severe neurological disability; follow-up: 29 years
Severinski et al., 2016 (31)	F	11 years	No	18 h	CT: diffuse edema; CT (72 hours later): cerebral herniation	Altered consciousness, dilated pupils, bradycardia, hypertension, GCS 12	Yes	Yes	No	7.005	mannitol	Death
Sudhanshu et al., 2016 (32)	F	13 years	Yes	4 h	NA	Altered consciousness, responsiveness only to deep pain, bradycardia, hypertension, GCS NA	Yes	No	Yes	6.9	mannitol	Complete recovery; follow-up: 5 years (no neurological sequelae)
Mozzillo et al., 2015 (30)	F	9 months	Yes	10–11 h	CT: reduced CSF spaces	Seizures, dilated pupils, GCS 5	Yes	Yes	No	7.27	NA	Complete recovery; follow-up: 5 years (no neurological sequelae)
Gee et al., 2015 (29)	M	4 years	No	NA	CT: sulcal effacement, ventricular narrowing, diffuse hypodensity	Breathing disorders, decreased responsiveness, lethargy, GCS 10	Yes – lethargy, decreased responsiveness	No	No	6.94	mannitol 0.5 g/kg, hypertonic saline 5 mL/kg	Complete recovery; follow-up: NA

T1D, type 1 diabetes mellitus; DKA, diabetic ketoacidosis; CT, computed tomography; MRI, magnetic resonance imaging; M, male; F, female; NA, not available; GCS, Glasgow Coma Scale; CSF, cerebrospinal fluid.

Several conclusions can be drawn from our review. First of all, the clinical presentation in these patients was generally nonspecific, leading to the conclusion that any concerning neurological deterioration or lack of neurological improvement in children treated with DKA should raise suspicion of CE. While some children were in very severe general condition, others presented only with mild symptoms. Their GCS score varied from 3 to 15. Importantly, hyperosmolar therapy should be initiated immediately based on clinical findings, without awaiting neuroimaging, which should primarily serve to support diagnosis or exclude alternative causes of neurological deterioration. Neuroimaging also plays an important role in the differential diagnosis. In patients with DKA who develop acute neurological deterioration during treatment, CE is the most likely diagnosis. However, other, less common neurological complications should also be considered. The available literature describes cases of cerebral venous sinus thrombosis, ischemic stroke, intracranial hemorrhage, and posterior reversible encephalopathy syndrome occurring during the course of DKA (42–45). In the absence of fever or elevated inflammatory markers, as in our patient, central nervous system infection is considered less likely. In such cases, lumbar puncture is contraindicated when increased ICP is suspected. Hyperosmolar therapy remains the recommended first-line treatment. In the reports summarized in **Table 2**, mannitol was the most frequently used agent, followed by hypertonic saline.

The available literature does not unequivocally define either the risk factors or pathophysiological mechanisms underlying DKA-related CE. Traditionally, the osmotic shift hypothesis proposed that rapid changes in serum osmolality during DKA treatment promote water movement into the brain, contributing to cerebral edema (6). Therefore, the avoidance of abrupt osmotic shifts, together with continuous monitoring of laboratory values, such as corrected sodium and effective osmolality, is crucial during treatment (46). However, increasing evidence indicates that cerebral hypoperfusion before treatment, followed by reperfusion injury, blood–brain barrier dysfunction, and neuroinflammatory processes may also play important roles (6, 9). Various studies suggest that multiple clinical and patient-related factors, including geographic, socioeconomic, and demographic characteristics, may influence the risk of this complication (6, 47, 48). Given the complexity of the pathophysiology of CE, it remains difficult to determine whether its development in our patient was primarily associated with the severity of DKA, treatment-related factors, or a combination of both. In most of the reports identified in our review, CE occurred in patients with new onset of diabetes, markedly elevated glucose levels, and severe metabolic acidosis characterized by low arterial pH, low initial bicarbonate concentration and reduced partial pressure of carbon dioxide. These features were also present in our patient, along with PICU admission, and bicarbonate administration, the latter being uncommon in most other reports.

## 4 Limitations

However, we acknowledge several limitations of our study. Due to the retrospective nature of the case description, some clinical data could not be retrieved from original medical records. Furthermore, the findings are limited to a single case report, and therefore, broader conclusions should be interpreted with caution. In addition, no formal, standardized developmental, school-performance, or neurocognitive assessment has been performed in our patient to date; such evaluation is recommended in similar future cases to more comprehensively assess potential long-term sequelae. Regarding the literature review, the number of published cases addressing this topic remains limited. Additionally, some of the included reports provided incomplete clinical data, as indicated in **Table 2**. Therefore, definitive conclusions regarding risk factors or causal relationships cannot be established, underscoring the need for further research. Moreover, the literature included in our analysis may be affected by publication bias, as cases with atypical or severe clinical presentations are more likely to be reported. Finally, the selection and data extraction process performed by a single reviewer represents an important limitation of our review.

## 5 Conclusions

Management of DKA and DKA-related CE in the pediatric population remains challenging. The presented case, together with the literature review, emphasizes the critical role of early and accurate diagnosis. Despite advances in DKA management, CE remains a life-threatening complication in pediatric patients. Careful management of fluid therapy, insulin administration, electrolyte and acid-base disturbances, together with continuous neurological monitoring, remains essential throughout treatment. Prompt recognition of neurological deterioration and immediate intervention, including administration of hyperosmolar agents when indicated, are crucial. Given that the mechanisms underlying CE are complex and not fully understood, further research is needed to clarify its pathogenesis, identify modifiable risk factors, and improve strategies for early recognition and prevention in children at highest risk.

## Data Availability

The original contributions presented in the study are included in the article/**Supplementary Material**. Further inquiries can be directed to the corresponding author.

## Supplementary material

Supplementary Audio S1 The patient’s characteristic Kussmaul breathing.
